# Top consumer abundance influences lake methane efflux

**DOI:** 10.1038/ncomms9787

**Published:** 2015-11-04

**Authors:** Shawn P. Devlin, Jatta Saarenheimo, Jari Syväranta, Roger I. Jones

**Affiliations:** 1 Department of Biological and Environmental Science, University of Jyväskylä, PO Box 35, 40014 Jyväskylä, Finland

## Abstract

Lakes are important habitats for biogeochemical cycling of carbon. The organization and structure of aquatic communities influences the biogeochemical interactions between lakes and the atmosphere. Understanding how trophic structure regulates ecosystem functions and influences greenhouse gas efflux from lakes is critical to understanding global carbon cycling and climate change. With a whole-lake experiment in which a previously fishless lake was divided into two treatment basins where fish abundance was manipulated, we show how a trophic cascade from fish to microbes affects methane efflux to the atmosphere. Here, fish exert high grazing pressure and remove nearly all zooplankton. This reduction in zooplankton density increases the abundance of methanotrophic bacteria, which in turn reduce CH_4_ efflux rates by roughly 10 times. Given that globally there are millions of lakes emitting methane, an important greenhouse gas, our findings that aquatic trophic interactions significantly influence the biogeochemical cycle of methane has important implications.

## Abstract (web summary)

How aquatic communities influence biogeochemical cycling is not well understood. Here, Devlin *et al.* manipulate the abundance of fish in a whole-lake experiment and show that methane efflux is reduced by the presence of top predators, via a trophic cascade from zooplankton to methanotrophic bacteria.

## Graphical abstract

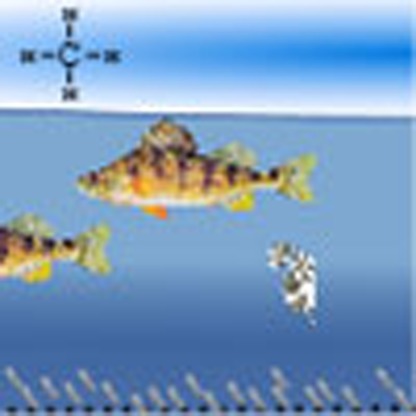

Lakes are hotspots of biogeochemical carbon cycling^1^,^2^ and the ecosystem functions that regulate carbon cycling are often mediated by trophic interactions^3^. Methanogenesis occurs extensively in anoxic sediment or hypolimnetic water in lakes^4^. Although emissions of methane (CH_4_) from lakes may be small relative to those of inorganic carbon (CO_2_), the nearly fourfold greater atmospheric warming potential of CH_4_ compared with CO_2_ means the overall importance to the global carbon cycle and potential impacts on global climate change are high^1^. The high dissolved organic carbon (DOC) concentrations and steep thermal stratification found in small humic lakes across the boreal zone lead to particularly high methanogenesis, water column concentrations of CH_4_ and potential efflux to the atmosphere^4^,^5^,^6^. However, where diffusing CH_4_ meets an anoxic–oxic interface, methane-oxidizing bacteria (MOB) can thrive and can extend throughout the oxygenated epilimnion. MOB can oxidize 30–99% of CH_4_ in the water column, thus regulating CH_4_ concentrations and potential emissions^7^. Energy mobilization via microbial communities is now recognized as an important parallel pathway to photosynthetic primary production in aquatic food webs^8^, and MOB are one group of microbes that can provide a link between DOC and aquatic consumers. Incorporation of methane–carbon into consumer biomass via grazing on MOB has been shown to contribute substantially to the carbon requirements of zooplankton, chironomid larvae and other zoobenthos and some fish^9^. Regulation of methane oxidation via trophic interactions and grazing by zooplankton might therefore be expected to be a key factor determining methane concentrations in lakes and hence methane emissions to the atmosphere.

Cascading trophic interactions have become a central tenet of food web ecology and are paramount in the conceptual understanding of trophic dynamics^10^. Top–down regulation of biomass and the subsequent release of successive trophic levels regulate trophic structure, biomass, and most importantly ecosystem processes such as respiration and primary production. Trophic cascades in lakes from fish via herbivorous zooplankton to phytoplankton have been particularly well documented, although cascading trophic interactions have been shown in many ecosystems^11^. In contrast, microbial community dynamics are more often considered to be driven primarily by ‘bottom-up' energy and nutrient limitation^12^,^13^. Firm evidence that trophic cascades can regulate ecosystem functions other than primary production or respiration is lacking. Predatory mites have been reported to increase N_2_O emissions from soil via regulation of fungivores, but it is unclear if this is due to trophic release or to detrital-based nutrient subsidy^14^.

To demonstrate that trophic cascades can regulate ecosystem functions, such as CH_4_ efflux to the atmosphere, several criteria must be met^11^,^15^. Under top–down trophic regulation, predation pressure must result in a decrease in prey. The prey's own food source must demonstrate a corresponding increase or lack of reduction in abundance and this increase/stasis must be accompanied by an increase in functional traits of the basal food source (that is, increased methanotrophy or reduced methane efflux)^16^. To study the possible impact of a trophic cascade on lake methane efflux, we partitioned Mekkojärvi, a highly humic lake in south central Finland, with a plastic curtain to create two discrete basins. As Mekkojärvi is naturally fishless because of complete winter under-ice anoxia, high densities of the crustacean zooplankter *Daphnia longispina* were present at the onset of the experiment. These *Daphnia* are known to graze extensively on MOB in the lake^17^,^18^. During the course of 3 years, early in each July we added European perch (*Perca fluviatilis*) to one of the two basins while the other remained fishless. We thereby created contrasting fish-present and fish-absent treatments, which we predicted would alter existing zooplankton densities accordingly. We predicted and demonstrate that the presence of fish decreases zooplankton abundance, which in turn, releases MOB from grazing pressure resulting in no reduction of MOB abundance with time. In the absence of fish, high grazing pressure by zooplankton decreases MOB abundance significantly over time. Further, we predicted and demonstrate that contrasting MOB abundances lead to concomitant differences in methane oxidation, and hence, both concentrations of CH_4_ in the upper water column and CH_4_ efflux from the lake to the atmosphere. However, the different trophic interactions in the oxic water column do not affect methanogenesis and methane concentrations in the anoxic hypolimnion (Fig. 1).

## Results

### Fish additions significantly lowered zooplankton abundance

**Figure 2:**
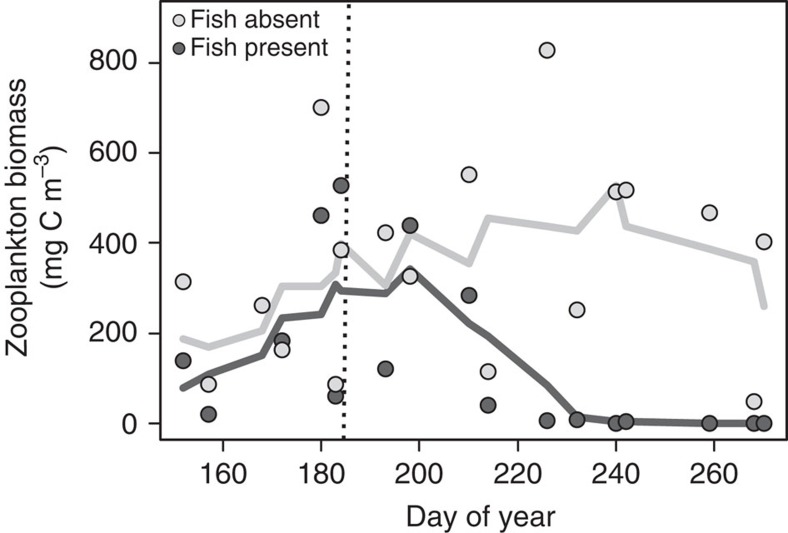
Introduction of fish reduced the zooplankton biomass each year during the whole-lake manipulation experiment. Each data point represents the measured zooplankton biomass (mg C m^−3^) from either fish-present or fish-absent treatment basins at different times through the ice-free season during the 3 years of manipulation (*n*=2 or 3 for each sampling event pooled across years). The solid lines represent the moving average of zooplankton abundance and the vertical dashed line represents the approximate date fish were added to the appropriate basin each year.

**Figure 3:**
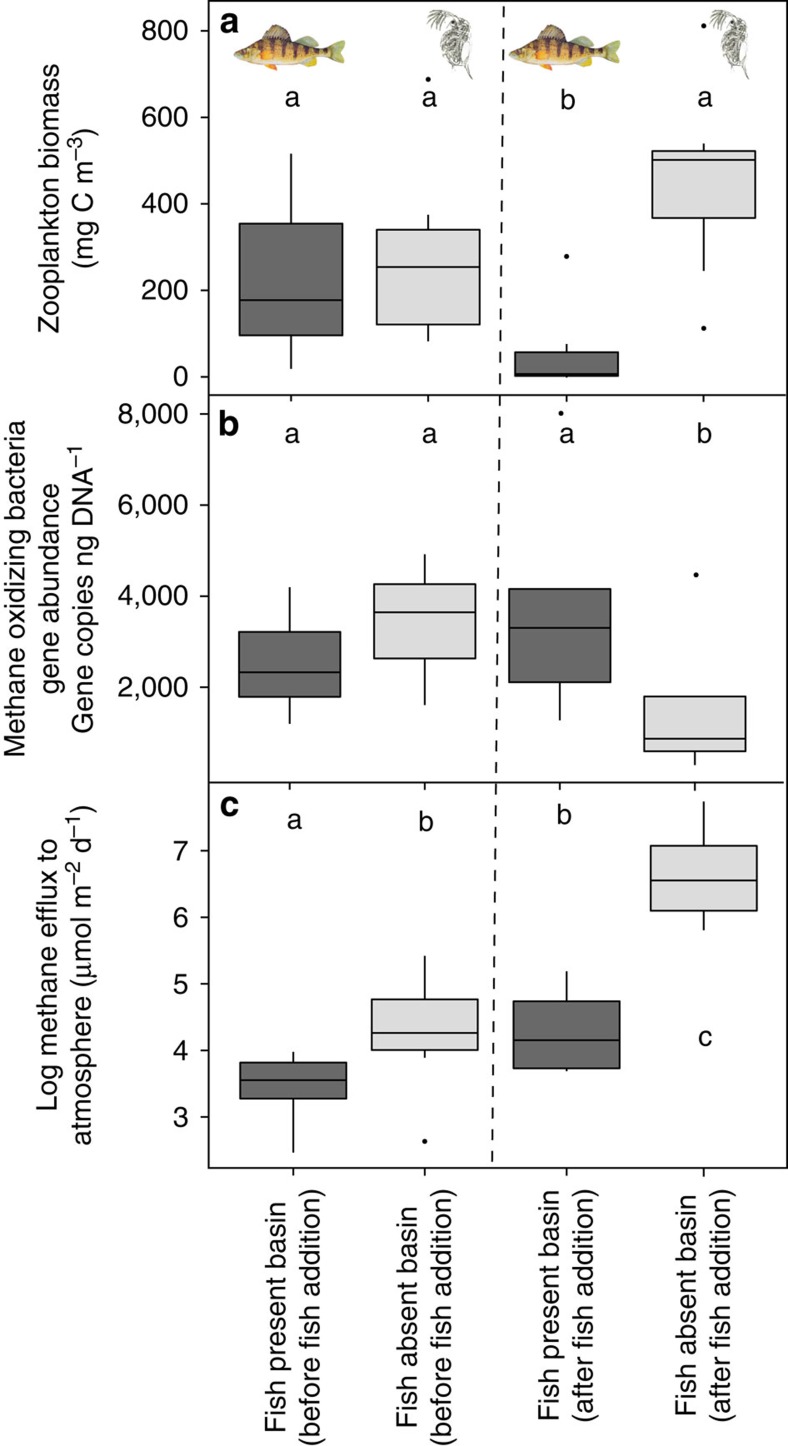
Fish presence affects zooplankton abundance, abundance of MOB and calculated methane efflux from lake to atmosphere. Box-and-whisker plots for the fish-present and fish-absent treatments for both the pre-fish period and the post-fish addition. The line across the box represents the median, the box edges represent the upper and lower quartile, and the whiskers represent the 95th percentile. Single data points are considered to be outside the 95th percentile, however, in most cases the data were not excluded from analysis. (**a**) Fish presence significantly decreased zooplankton abundance over time (Welch *t*-test: *t*=3.2157, d*f*=8, *P*=0.01) whereas when fish were absent there was no change in abundance with time observed (Welch *t*-test: *t*=−1.1822, d*f*=8, *P*=0.26) and significantly higher zooplankton abundance (Paired *t*-test: *t*=−4.449, d*f*=8, *P*=0.0030). (**b**) A significant decrease in MOB gene abundance over time is seen in the absence of fish (Welch *t*-test: *t*=4.543, d*f*=9, *P*=0.0016) while there is no significant change in MOB gene abundance when fish were present (*P*>0.05) leading to a significant difference between the treatment basins (Paired *t*-test: *t*=4.1639, d*f*=4, *P*=0.0141). (**c**) A significant seasonal increase in methane efflux from lake to atmosphere occurs under both fish presence (Welch *t*-test: *t*=−2.4134, d*f*=9, *P*=0.0448) and absence treatments (Welch *t*-test: *t*=−4.9689, d*f*=9, *P*<0.001). However, the significant difference between basins (Paired *t*-test: *t*==4.062, d*f*=6, *P*=0.027) is a striking 9.9 times more efflux when fish are absent compared with only a 150% increase when fish are present.

The presence of fish had a significant impact on zooplankton density, drastically reducing the biomass to nearly zero in the fish-present treatment basin (Figs 2 and 3a). Before the addition of fish there was no difference in zooplankton abundance between treatment basins (Paired *t*-test: *t*=−1.4763, d*f=*8, *P*=0.1781). After fish were added, zooplankton biomass was significantly lower in the fish-present basin relative to the basin with no fish (Paired *t*-test: *t*=−4.449, d*f*=8, *P*=0.003) and zooplankton biomass significantly decreased within the basin after the fish addition (Welch *t*-test: *t*=−3.2157, d*f*=8, *P*=0.0123). In contrast, there was no significant difference within the fish-absent treatment basin before and after the date of the addition of fish (Welch *t*-test: *t*=−1.1822, d*f*=8, *P*=0.2572). The zooplankton community was dominated by *Daphnia*, which comprised roughly 85% of the total community. The genus *Polyphemus* represented nearly all the remaining 15% of the zooplankton community with only a few individual *Chaoborus* and *Cyclopoida* found each year.

### Fish additions significantly affected MOB gene abundance

During the pre-fish addition period abundance of MOB, as indicated by their gene abundance, was similar between treatment basins (Paired *t*-test: *t*=−1.0173, d*f*=7, *P*=0.343; Fig. 3b). While there was no observed temporal change in MOB gene abundance when fish were present (Welch *t*-test: *t*=−1.0007, d*f*=4, *P*=0.36), there was a significant reduction over time when fish were absent (Welch *t*-test: *t*=−4.4543, d*f*=9, *P*=0.016) leading to a profound 266% more MOB genes in the fish-present basin after the addition of fish (Paired *t*-test: *t*=4.1639, d*f*=4, *P*=0.0141).

### Fish additions significantly affected methane efflux

The addition of fish lead to a significant and large difference in CH_4_ efflux between treatment basins (Paired *t*-test: *t*=−4.062, d*f*=4, *P*=0.016) and reduced methane emissions by roughly 10 times (Fig. 3c). Although the basins were significantly different before fish additions (Paired *t*-test: *t*=−2.9041, d*f*=6, *P*=0.027), the fish-absent basin had only 164% more efflux than the fish-present basin, whereas after the effect of fish it was 989% higher. The natural temporal increase in CH_4_ efflux with time is demonstrated by the significant increase in efflux before and after fish were added to the fish-present treatment basin (Welch *t*-test: *t*=−2.4134, d*f*=9, *P*=0.049) and constituted a 150% increase in efflux over time when fish were present relative to an increase of 932% when fish were absent.

### Fish additions had no effect on methanogenesis

**Figure 4:**
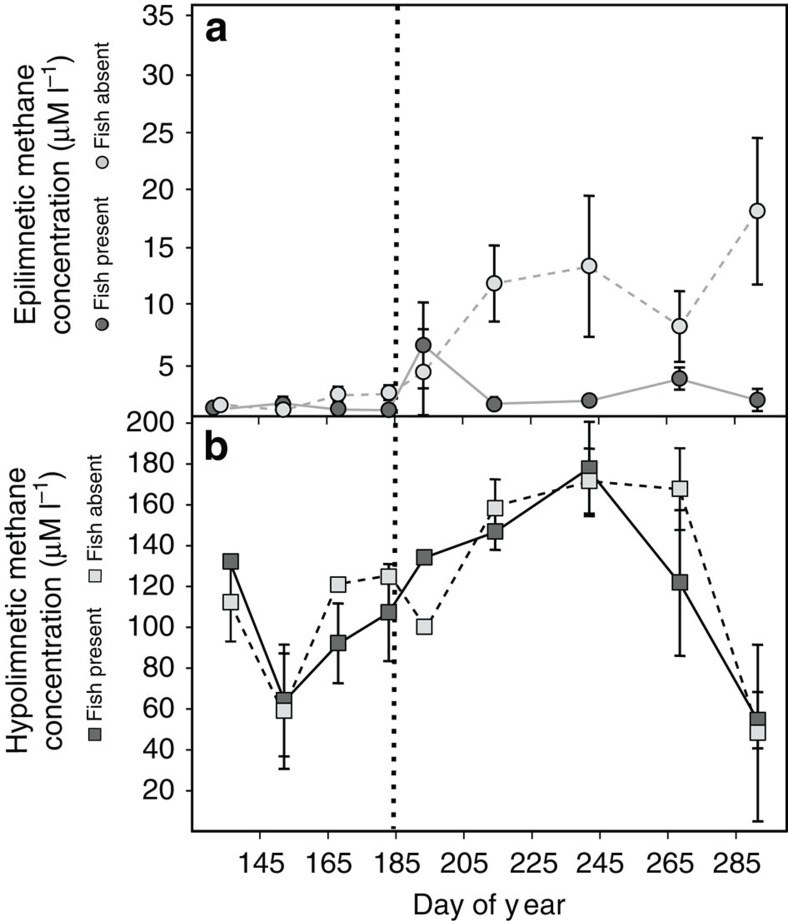
Methane (CH_4_) concentrations in the epilimnion (a) and hypolimnion (b) of each treatment basin through the ice-free seasons of the 3 years of manipulation. Data points are means (±s.e.) from the 3 years of the whole-lake manipulation (*n*=3). The vertical dashed line represents the approximate date fish were added to the appropriate basin. The epilimnetic CH_4_ concentrations between basins become significant different after the fish additions (Paired *t*-test: *t*=−18.508, d*f*=3, *P*=0.03). There was no significant difference in hypolimnetic CH_4_ concentration between treatment basins before or after fish were introduced (Paired *t*-test: *t*=−0.6262, d*f*=4) indicating fish had no effect of methanogenesis. Hypolimnetic CH_4_ concentrations are on average 140 × greater than epilimnetic CH_4_ concentrations indicating high methanogenesis in the anoxic hypolimnion.

The concentration of CH_4_ in the epilimnion was significantly different between basins after the fish addition (Paired *t*-test: *t*=−18.508, d*f*=3, *P*=0.025), whereas no difference existed before fish were added (Paired *t*-test: *t*=−0.0706, d*f*=3, *P*=0.9471; Fig. 4a). The hypolimnetic CH_4_ concentrations were similar and no significant differences were observed between basins before (Paired *t*-test: *t*=−0.249, d*f*=4, *P*=0.82) or after (Paired *t*-test: *t*=−0.6262, d*f*=2, *P*=0.5951) fish additions (Fig. 4b), demonstrating that methanogenesis was equal across basins and through time and not changed by the presence of fish.

## Discussion

Top–down control exerted by fish had profound and striking impacts on zooplankton density, MOB abundance and methane efflux. Zooplankton were abundant in the absence of fish, whereas zooplankton biomass was clearly reduced after addition of fish (Fig. 2). Such effects of fish on zooplankton have been extremely well documented as being widespread in lakes of all kinds^10^,^19^. Stable isotope and fatty acid analyses have shown that zooplankton in Mekkojärvi feed extensively on MOB^17^,^18^ and hence are expected to exert a top–down pressure on MOB abundance. Carbon stable isotope analysis of zooplankton during 2013 confirmed *Daphnia* grazing on MOB during our lake manipulation (Supplementary Fig. 1). The reduction in zooplankton biomass following the addition of fish released MOB from grazing pressure and allowed MOB to remain abundant (Fig. 3a). The significant decrease in MOB gene frequency when the basin remained fishless and the lack of a corresponding decrease when fish were present demonstrate causal trophic dynamics and not simply natural temporal patterns in bacterial community composition or abundance. Hence, our results clearly illustrate that a trophic cascade from fish extends as far as microbes. As a consequence of the higher abundance of MOB when fish are present relative to when fish are absent, CH_4_ concentration in the epilimnion was lower after fish introduction (Fig. 4a), reflecting the high MOB abundance and associated relatively higher oxidation of methane. In contrast, trophic interactions in the oxic layer of the water column had no effect on CH_4_ production, as reflected by hypolimnetic CH_4_ concentration (Fig. 4b). Methane production in lakes can be spatially heterogeneous as redox conditions, depth and bacterial communities vary around a lake^4^,^20^. However, we found no significant difference in mean hypolimnetic CH_4_ concentrations between the two experimental basins and therefore conclude that the observed significant difference in mean epilimnetic CH_4_ concentration between the two lake basins was due to the influence of the trophic cascade down the food web.

A clear temporal pattern in calculated methane efflux was apparent, with values being lower early in the year (when both experimental basins lacked fish) than later in the year during the period after fish had been introduced to one basin (Fig. 3c). However, although there was a significant increase in CH_4_ efflux after fish addition in the fish-present treatment, this increase was nearly two orders of magnitude less than the temporal increase when fish were absent (Fig. 3c). The temporal pattern of methane efflux observed for both basins reflects the seasonal accumulation of CH_4_ in hypolimnetic water (Fig. 4b) throughout the course of the ice-free season up to the onset of fall overturn of the water column. A slow but steady upward diffusion of this methane to the epilimnion leads to higher concentrations there and increased efflux to the atmosphere. However, this seasonal increase is small (∼150%) when fish are present because the abundant MOB can oxidize most of the upwardly diffusing CH_4_. In contrast, when fish are absent the sparse MOB pool is only able to oxidize a small part of the upwardly diffusing CH_4_ so that a high proportion reaches the surface and evades to the atmosphere. These results demonstrate a clear influence of fish on methane dynamics in the lake.

One possible response that we did not measure and was not included in this study was a potential reciprocal increase in non-Daphnia bacterivores (that is, rotifers, protists, ciliates and heterotrophic nanoflagellates). These organisms would have been released from competition with and/or grazing pressure from crustacean zooplankton and likely increased in abundance and continued to graze MOB to some degree. Anecdotally, while counting crustacean zooplankton, rotifers were observed throughout the ice-free season and within each treatment basin and, qualitatively, no obvious change in abundance in response to fish presence was observed. There could have been an increase in protozoan numbers and some concurrent grazing of MOB, but we have no evidence of this as we did not count these groups. Although protozoans are considered to be major aquatic bacterivores^21^,^22^, the overwhelming response of MOB gene abundance and CH_4_ efflux to the presence of fish indicates that, in Mekkojärvi, it is only the large bodied *Daphnia* that exert strong top–down control of large MOB cells. To qualify as a trophic cascade, trophic interactions must have alternating effects on biomass or function at each subsequent trophic position^16^. Here we show that the presence of fish has a strong effect on zooplankton biomass and the underlying foundational trophic level. This has been demonstrated previously and frequently for the case of a trophic cascade from fish down to autotrophic phytoplankton^11^. However, to our knowledge, our results are the first demonstration of a trophic cascade from top consumers affecting microbial communities and an associated biogeochemical cycle^23^. A previous marine study described a trophic cascade affecting bacterial numbers but did not illustrate an effect on microbial ecosystem functioning^24^.

Trophic cascades do have the potential to regulate ecosystem function at lower trophic levels. Fish presence in mesocosms was shown to increase out-gassing of CO_2_, presumably as heterotrophic bacteria were relieved from zooplankton grazing pressure leading to increased respiration of organic matter cycled through the microbial loop^25^. Similarly, in a whole-lake manipulation experiment, fish increased utilization and sequestration of CO_2_ via a cascade of grazing pressure leading to reduced zooplankton grazing on phytoplankton^3^. Our results are analogous to these studies, but for the first time extend the concepts to energy mobilizers in aquatic food webs that are not part of the core autotrophic pathway. We speculate that CH_4_ oxidized via MOB activity is either incorporated into microbial biomass and potentially passed up the food web or is shunted into the dissolved inorganic carbon (DIC) pool from where potentially it can be off-gassed as CO_2_. This may result in no net change in the mass of carbon emitted to the atmosphere over the long term, but the form of carbon, and thus its potential warming effect as a greenhouse gas, is dramatically changed. Carbon dioxide is far less potent than CH_4_ as a greenhouse gas^1^ leading to a substantial reduction in the warming effect of lake emssions when fish are present.

Our study presents the first evidence of macroconsumer regulation of bacterial abundance and an associated biogeochemical cycle in freshwater ecosystems via a trophic cascade, and demonstrates a clear link between trophic structure and lake contributions to atmospheric greenhouse gas pools. In the presence of fish, methane efflux from the lake to the atmosphere was reduced to around one tenth. Mekkojärvi provided a model system for ‘proof-of concept' that top consumer abundance can influence methane dynamics in lakes through a trophic cascade. The results presented here are a demonstration that trophic interactions can play a role in regulating biogeochemical cycles. However, the complete lack of fish and subsequent fish introduction, and the almost complete lack of zooplankton represent contrasting extremes of top–down trophic control. In practice, most boreal lakes will fall between these two extremes and will represent a dynamic equilibrium in regard to trophic regulation. Hence our evidence of top–down regulation of methane efflux reported here cannot be extrapolated indiscriminately to other lakes. However, on a global scale there are more than 2.9 × 10^7^ lakes of similar size to Mekkojärvi^5^. Especially across the boreal zone, many of these lakes are strong CH_4_ emitters, and CH_4_ emissions from lakes have been estimated to contribute as much as 8–48 Tg per year (6–16%) to the global natural CH_4_ emissions^4^. Given that CH_4_ is 25 times more active as a greenhouse gas than CO_2_ in a time horizon of 100 years^26^, our finding that CH_4_ emissions from lakes can be influenced by changes in consumer abundance at the top of lake food webs has implications for understanding lake ecosystems as integral components of carbon cycling in the landscape. Many small lakes like Mekkojärvi in high northern latitudes are naturally fishless because of long winter ice cover. With climate warming the duration of ice cover will decrease while many fish species are extending their distributions northwards^27^. Our findings suggest that these changes can have appreciable and previously unsuspected implications for methane dynamics in lakes and the atmosphere.

## Methods

### Study site lake division and fish addition

Mekkojärvi (61° 13′ N, 25° 8′ E), is a small, shallow lake (area 0.35 ha, mean depth 3 m) in the Evo forest area of southern Finland that has a strong history of limnological research (see Supplementary Table 1 for lake characteristics). The lake is surrounded by dense forest and abandoned agricultural land and is highly humic, with average DOC concentrations between 25 and 35 mg C l^−1^. The small size and brown water result in steep thermal and oxygen stratification. The lake is ice-free usually from the beginning of May to mid-November, and the whole water column turns over in autumn but only partially in spring. During summer only the uppermost 0.5–1 m layer is oxic. Anoxic conditions in the sediment and overlying hypolimnetic water in the presence of large amount of organic matter provides ideal habitat for methanogenic bacteria supplying the water column with large amounts of methane (CH_4_). Further, fall overturn mixes the relatively large volume of anoxic hypolimnion with the smaller volume of oxic epilimnion, which leads to strong oxygen depletion through the entire water column. Complete water column anoxia rapidly develops and persists under the long winter ice cover leaving the lake naturally fishless and providing a suitable template for whole-lake experimental manipulations focusing on trophic interactions.

The lake was divided with a ∼100 m solid plastic curtain that was suspended by a rope attached tautly to two trees at opposite sides of the shoreline. The curtain was fitted to the bathymetry of the lake with enough material to ensure the entire water column would be divided. The bottom of the curtain was weighted with sand to ensure that it seated into the soft lake bottom sediments. The curtain was lifted each fall before ice developed to allow complete mixing between the treatment basins during the winter and early spring to allow re-establishment of zooplankton and to reset any biogeochemical changes that resulted from the previous year separation.

In early July of each year, European perch (*Perca fluviatilis* L.) were added in numbers representing naturally occurring perch biomass in similar lakes in the Evo forest area (∼1 g m^−2^ of lake surface area ) in such a way as to establish fish-present and fish-absent treatments each year between 2011 and 2013. Permission to carry out the fish additions in Mekkojärvi was obtained from the local landowner, and the introductions were carried out with the approval of and in conjunction with the Finnish Game and Fisheries Research Institute. In 2011 ∼100 adult perch (>12 cm) were added to one basin and 800 juvenile (<3 cm) young of year individuals were added to the other basin. However, due to a brief pulse of hypoxic conditions in one basin the adults died shortly after introduction leaving the basin fishless. In 2012, the same treatments were applied, but brief hypoxia occurred following heavy rains and the young of year fish perished leaving an adult population in one basin and no fish-present in the other basin. This occurred again in 2013. The duration of the hypoxia was brief, but because the introduced fish were recently caught and transported they could have been particularly susceptible to brief reductions in oxygen. Given the short time frame of hypoxia and the integrated nature of the biogeochemical processes at play, it is unlikely that this event had strong impacts on our findings. Thus the pattern of fish additions and mortality in the basins effectively yielded two treatments, fish-present and fish-absent, which led to high and low planktivory, respectively, and the zooplankton biomass responded accordingly.

### Zooplankton biomass and bacterial sampling

Water samples for zooplankton biomass and bacterial DNA extraction were collected and pooled from epilimnion (0–0.5 m), metalimnion (0.5–1 m) and hypolimnion (1–3 m). Zooplankton was collected with a Limnos water sampler by taking 6 l of sample water from epi-, meta- and hypolimnion, and filtering the samples through a 50-μm mesh net. The samples were later identified, counted and measured under stereo microscopes. *Daphnia* biomass (*w*, mg C) was then calculated using length–weight relationships *w=aL*^*b*^ from Rahkola *et al.*^28^ where intercept *a* was estimated at 5.66, *L* is the *Daphnia* body length in mm and slope *b* was 1.72. This process was repeated throughout the experiment for each stratum in each basin fortnightly or monthly depending on time of year. For bacterial measurements, 100 ml of the water that passed though the zooplankton net from each stratum and sampling event was reserved and immediately frozen.

### DNA extraction and qPCR of 16S rRNA and *pmoA* genes

The bacteria samples were lyophilized (Alpha 1–4 LD plus, Christ GmbH, Osterode am Harz, Germany) and extracted with PowerSoil DNA extraction kit (Mo Bio Laboratories, Inc., Carlsbad, California, USA) according to the manufacturer's instructions. After extraction, DNA concentrations of the samples were measured with Qubit 2.0 Fluorometer (Invitrogen Life Technologies, Grand Island, NY, USA).

Amplification of quantitative PCR (qPCR) and fluorescent data collection was carried out with a Bio-Rad CFX96 thermal cycler (Bio-Rad Laboratories, Hercules, California, USA) in a reaction mixture of 20 μl containing 0.5 μM of each primer for selected gene, 10 μl 2XiQ SYBR Green supermix (BioRad) and 1 μl of DNA and PCR-grade water (Fermentas, ThermoFisher, Waltham, MA USA). To amplify most of the methanotrophs and to achieve best results, we tested several different primer pairs used in previous studies and selected those copying the highest number of methanotrophs, listed in Supplementary Table 2.

The PCR amplification was done as described previously for *pmoA* (ref. 29) and for 16S rRNA (ref. 30), with slight modifications in the PCR procedure. Briefly, the amplification procedure for 16S rRNA included an initial denaturation step at 95 °C for 15 min and 40 cycles of amplification (95 °C for 25 s, 53 °C for 35 s and 72 °C for 45 s). Finally, an increase of 0.5 °C s^−1^ from 65 to 95 °C was added to obtain the melting curve analysis. The thermal cycling conditions for other genes were otherwise similar except that the annealing temperature was 54 °C for MBAC assay and 60 °C for MCOC assay. Information on primer sets can be found in Supplementary Table 2. Standard curves were constructed from PCR amplicons extracted from agarose gel with BioRad Gel Extraction Kit (BioRad). Amplicons were re-amplified using PCR and the PCR products were purified with Agencourt AMPure XP (Beckman Coulter, Brea, California, USA). In each qPCR run, a dilution series of 10^7^–10^2^ gene copies were used as quantification standards. Inhibition was tested from the dilution series (10^0^, 10^−1^ and 10^−2^) showing no effect on the PCR efficiency.

### Zooplankton stable isotope values

Samples of *D. longispina* for carbon stable isotope analysis were collected twice per month in 2013 from May until fall turnover in October using 50 and 100 μm mesh nets towed horizontally at different depths. The samples were then quickly transported to the laboratory, sorted visually, and left alive in clean tap water overnight for *Daphnia* to empty their guts. Samples were then filtered and put into 2 ml glass vials or Eppendorf tubes, and were dried in an oven at 60 °C. Approximately 0.5 mg of dried sample material was accurately weighed into tin cups for analyses of δ^13^C at the University of Jyväskylä using a FlashEA 1112 elemental analyser coupled to a Thermo Finnigan DELTAplus Advantage mass spectrometer (Thermo Electron Corporation, Waltham, MA, USA).

### Nutrients

Concentrations of total dissolved inorganic P (PO_4_^3−^), N (NH_4_^+^+NO_3_^−^) and DOC of lake water were determined following standard laboratory methods ( http://www.sfs.fi/). Samples were collected with a Limnos water sampler and brought to the laboratory on ice. Nutrient samples were then filtered through Whatman GF/C glass fibre filters and kept frozen until the analyses. DOC concentrations were measured from water filtered through Whatman GF/F filters and samples were similarly kept frozen until the analyses.

### Methane concentration and efflux

Replicated water samples (30 ml) for headspace analysis of CH_4_ concentrations were taken from surface, epi-, meta- and hypolimnion. Samples were taken in 60 ml polypropylene syringes with three-way stopcocks, which were closed after carefully removing any gas bubbles from the syringes. The syringes were then kept in crushed ice and transported to the laboratory, where the syringes were warmed to 20 °C and 30 ml of N_2_ gas was inserted through the three-way stopcocks. The syringes were then put into a shaker to extract the dissolved CH_4_ followed by injection of sample gases into 12 ml exetainers (Labco Limited, Ceredigion, UK) and CH_4_ analysis with an AGILENT 6890N (Agilent Technologies) gas chromatograph equipped with a FID detector^31^.

The boundary layer diffusion equation presented by Kling *et al.*^32^ and Phelps *et al.*^33^ was used to estimate the methane effluxes from Mekkojärvi during the experiment: CH_4_ efflux=D_b_/z_b_ × (C_sur_−C_eq_), where C_sur_ is the concentration of CH_4_ measured in the epilimnion (0–50 cm depth), C_eq_ is the concentration of CH_4_ in equilibrium with air, z_b_ is the thickness of the boundary layer and D_b_ is the diffusion coefficient. Db (cm^2^ s^−1^) and z_b_ (μm) were calculated with the following equations: D_b_=(1.33+(0.055 × T)) × 10^−5^ and zb=10^(2.56−(0.133 × ws)^, where T is the water temperature (°C) at the surface and ws is wind speed at 10 m height (m s^−1^). The wind speed is an average wind speed measured between years 2002 and 2009 at a nearby lake Valkea Kotinen^34^,^35^, which presents a similar kind of environment and where the wind conditions well represent the conditions at Mekkojärvi. The C_eq_ was calculated with Henry's law constants for surface temperatures^36^ assuming a stable atmospheric CH_4_ concentration of 1.745 p.p.m. (ref. 37).

### Data analysis

A series of Welch *t*-tests and Paired *t*-tests were conducted to assess if significant changes in the zooplankton abundance, MOB gene abundance and CH_4_ efflux occurred after the addition of fish within and between basins. Data from the stratified period of each year were pooled to replicate the treatment effects resulting in a pooled sample size of three replicates for each sampling event. Data were then binned into pre-fish, transition, or post-fish periods and *t*-tests between pre-fish and post-fish periods were conducted (Supplementary Table 3). It should be noted that the transitional data for all variables were not included in statistical tests because in all cases it was not significantly different from the pre-fish or post-fish periods and invariantly lay intermediate to the means of the other periods. The pre-fish period was defined as from the development of stratification to the Fish additions. The Transition period was defined as the date of fish addition until zooplankton abundance leveled off near its minimum, ∼4 weeks after fish addition and the post-fish period lasting for the remainder of the stratified season. Statistical tests were considered significant when the *P* value was ≤0.05. Data collected while the lake was mixed or when the metalimnion was anoxic and extreme outliers were excluded from analysis. Owing to high and unequally distributed variance, CH_4_ efflux values were log transformed before analysis. All statistical tests were conducted in R (Ver. 3.1.2).

## Additional information

**How to cite this article:** Devlin, S. P. *et al.* Top consumer abundance influences lake methane efflux. *Nat. Commun.* 6:8787 doi: 10.1038/ncomms9787 (2015).

## Supplementary Material

Supplementary Information Supplementary Figure 1, Supplementary Tables 1-3 and Supplementary References

## Figures and Tables

**Figure 1:**
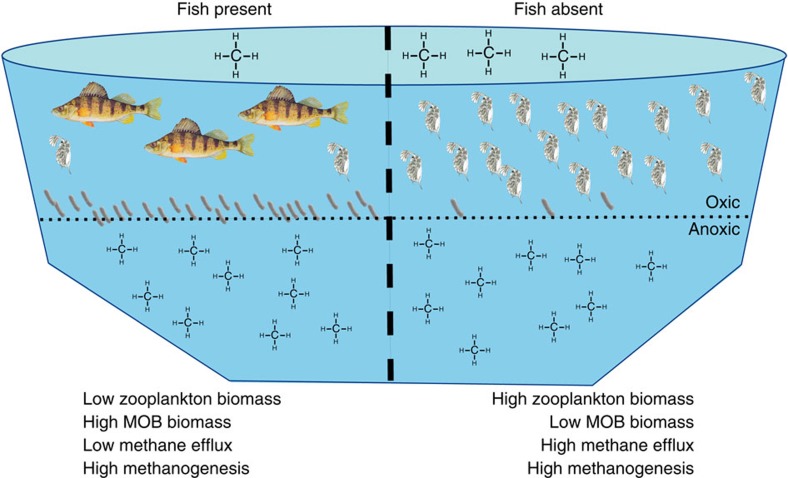
Conceptual schematic of the hypothesized effects of a trophic cascade from fish to microbes regulating methane efflux. A small stratified lake with an anoxic hypolimnion was divided into two basins with fish present (left) or absent (right). In the absence of fish, large populations of zooplankton graze strongly on MOB and reduce their abundance. This decreases potential methanotrophy leaving more methane available to escape from the lake to the atmosphere. Fish presence reduces zooplankton populations, releasing MOB from grazing pressure and leading to higher potential for methanotrophy relative to when fish are present. This results in less release of methane to the atmosphere from the lake. These trophic interactions in the oxic water column have no effect on methanogenesis occurring in the anoxic water and sediments.
