# Supplementary material for: Top consumer abundance influences lake methane efflux
Source: Nat Commun. 2015 Nov 4;6:8787. doi: 10.1038/ncomms9787 (PMC4659926; doi:10.1038/ncomms9787)
Supplement: Supplementary Information — Supplementary Figure 1, Supplementary Tables 1-3 and Supplementary References [file ncomms9787-s1.pdf]

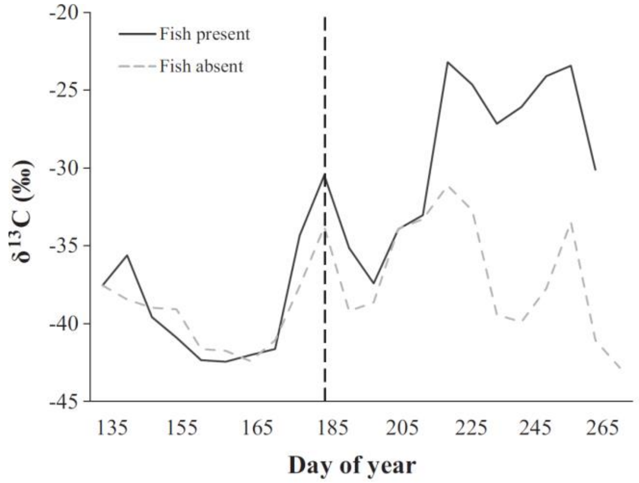

Supplementary Figure 1. Seasonal changes in *Daphnia* carbon stable isotope values in the two experimental basins during 2013. The vertical dashed line indicates when fish were added to one of the basins. ^{13}C-enriched bicarbonate was being added equally to both basins at intervals of a few days as part of a parallel experiment to label the phytoplankton at the base of the autotrophic food web, and this accounts for the irregularities in *Daphnia* $\delta^{13}C$ values. Following addition of fish to one basin the biomass of *Daphnia* in that basin became very low and the zooplankton evidently fed mainly on ^{13}C-labeled phytoplankton giving them higher $\delta^{13}C$. In the basin in which fish remained absent the abundant *Daphnia* used a variety of food sources including MOB. As MOB have very low $\delta^{13}C$ values, the *Daphnia* $\delta^{13}C$ values were also low, especially at fall turnover when production of MOB and their contribution to *Daphnia* diets becomes most pronounced[1,2].

Supplementary Table 1. Chemical characteristics by treatment basin, stratum and year for Mekkojärvi. Each value represents the ice-free season mean. Any measurement of DO below

0.6 mg L^{-1} was considered anoxic. Mean seasonal values are presented for each treatment basin (Fish Present and Fish Absent) for both the period before and after the additional of fish into the fish present basin.

Year	Before / After Fish Addition	Layer	Treatment basin	DO mg L^{-1}	Temp ºC	pH	DOC mg L^{-1}	NH4 µg L^{-1}	N-NO2/ NO3 µg L^{-1}	tot_P µg L^{-1}
2011	Before	Epi	Fish Present	5.17	15.10	-	27.70	30.00	35.50	7.50
		Epi	Fish Absent	4.28	14.68	-	26.03	18.50	96.50	9.00
		Meta	Fish Present	0.85	7.70	-	25.19	40.00	99.00	8.00
		Meta	Fish Absent	0.40	7.40	-	27.13	16.00	92.50	9.00
		Hypo	Fish Present	0.34	4.69	-	29.63	428.00	99.50	46.50
		Hypo	Fish Absent	0.31	4.60	-	30.77	428.00	68.50	51.00
	After	Epi	Fish Present	4.87	16.31	5.30	15.97	14.00	43.75	6.75
		Epi	Fish Absent	3.73	16.78	5.60	22.36	19.75	53.75	9.50
		Meta	Fish Present	1.60	11.83	5.70	20.43	15.00	43.00	8.75
		Meta	Fish Absent	0.53	11.98	5.80	27.53	73.00	47.50	17.00
		Hypo	Fish Present	0.49	6.63	5.80	28.51	745.50	43.25	116.25
		Hypo	Fish Absent	0.33	6.60	5.30	30.64	552.75	44.50	85.75
2012	Before	Epi	Fish Present	7.09	13.43	4.80	32.81	26.83	23.33	9.67
		Epi	Fish Absent	4.67	13.68	5.00	27.40	19.25	61.75	11.00
		Meta	Fish Present	3.61	6.47	5.40	32.73	38.33	47.50	9.33
		Meta	Fish Absent	0.81	6.23	5.20	33.36	31.00	50.50	12.50
		Hypo	Fish Present	0.60	4.64	6.10	28.78	546.00	26.33	80.33
		Hypo	Fish Absent	0.70	4.06	6.00	29.33	500.00	27.50	62.00
	After	Epi	Fish Present	5.28	14.08	4.95	33.61	28.50	39.17	11.42
		Epi	Fish Absent	4.18	14.38	5.27	34.76	33.67	22.50	13.16

		Meta	Fish Present	1.14	10.07	5.16	32.08	29.50	52.83	10.27
		Meta	Fish Absent	0.45	9.25	5.33	34.64	84.67	63.83	25.87
		Hypo	Fish Present	0.49	6.17	5.57	28.75	220.50	71.67	37.03
		Hypo	Fish Absent	0.57	5.94	5.59	32.28	256.00	118.33	45.60
2013	Before	Epi	Fish Present	5.10	17.10	4.48	35.37	50.00	37.00	13.33
		Epi	Fish Absent	2.65	17.43	5.25	31.15	29.00	51.67	11.00
		Meta	Fish Present	1.04	8.35	4.78	35.26	38.00	76.33	12.00
		Meta	Fish Absent	0.45	7.75	5.42	32.91	43.67	69.67	11.67
		Hypo	Fish Present	0.53	5.05	5.83	28.40	409.67	76.67	66.00
		Hypo	Fish Absent	0.56	4.80	5.83	31.61	396.00	84.00	58.00
	After	Epi	Fish Present	5.06	14.77	5.00	38.16	56.67	35.67	13.67
		Epi	Fish Absent	3.11	15.10	5.49	34.78	43.00	43.00	13.67
		Meta	Fish Present	0.67	10.10	5.17	31.47	39.67	70.67	14.67
		Meta	Fish Absent	0.30	9.63	5.51	38.13	65.00	67.67	23.00
		Hypo	Fish Present	0.33	6.39	5.95	27.90	422.00	65.67	70.33
		Hypo	Fish Absent	0.34	6.38	5.92	30.14	427.00	48.33	76.00

Supplementary Table 2. Primer pairs tested and used in the qPCR amplifying 16S rRNA and methanotroph *pmoA* and *mmoX* genes

Assay	Target group	Primers	Sequences	Reference	Annealing temp (C°)	Selected in the study
**16S rRNA**	16S rRNA	27f<br>338r	5’ AGAGTTTGATCNTGGCTCAG 3’<br>5’ -TGCTGCCTCCCGTAGGAGT-3’	[3]Universal primer	52	x
**MCOC**	*Methylococcus* group (*pmoA*)	A189F<br>Mc468R	5’-GGNGACTGGGACTTCTGG- 3’<br>5’-GCSGTGAACAGGTAGCTGCC-3’	[4]	60	x
**MBAC**	*Methylobacter/Methylosarcina* group (*pmoA*)	A189F<br>Mb601R	5’-GGNGACTGGGACTTCTGG- 3’<br>5’-ACRTAGTGGTAACCTTGYAA-3’	[4]	54	x
**Type II**	*Methylosinus* group (*pmoA*)	II233 F<br>II646 R	5’-CGTCGTATGTGGCCGAC-3’<br>5’-CGTGCCGCGCTCGACCARGYG-3’	[4]	69.5	
**MTOT**	*Methylobacter/Methylosarcina, Methylociccus, Methylosinus* group, *Methylocapsa, Nitroscoccus* (*pmoA*)	A189F<br>Mb661R	5’-GGNGACTGGGACTTCTGG-3’<br>5’-CCGGMGCAACGTCYTTACC-3’	[4]	60	

**mmoX**	*Methylocella* (*mmoX*)	mmoXLF<br>mmoXLR	5’-GAAGATTGGGGCGGCATCTG-3’<br>5’-CCCAATCATCGCTGAAGGAGT-3’	5	67
**Verruco microbial**	*Verrucomicrobia* (pmoA)	V170F<br>V613B	5’-GGATWGATTGGAAAGATMG-3’<br>5’-GCAAARCTYCTCATYGTWCC-3’	6	56

Supplementary Table 3. Raw data used to assess the effect of the presence of fish on zooplankton biomass, methanotrophic bacteria abundance, and methane efflux to the atmosphere during a whole-lake manipulation experiment. Dashes indicate missing data.

Date	Treatment	Period	Daphnia Biomass (mg C m^{-3})	MOB Abundance (gene copies ng DNA^{-1})	CH4 Efflux (μmol m^{-2} d^{-1})
5/16/2011	Fish	Mixed	147.1	1504	15.5
6/29/2011	Fish	PreFish	461.9	2041	27.4
7/12/2011	Fish	Trans	121.2	4931	278.3
8/2/2011	Fish	PostFish	41.2	8059	117.0
8/30/2011	Fish	PostFish	3.5	2155	65.7
9/27/2011	Fish	Mixed	0.7	8331	209.0
10/18/2011	Fish	Mixed	0.0	16597	34.2
5/16/2012	Fish	Mixed	6.8	188	26.1
5/31/2012	Fish	PreFish	139.1	2975	36.4
6/20/2012	Fish	PreFish	183.1	2375	47.9
7/2/2012	Fish	PreFish	528.0	1240	55.3
7/16/2012	Fish	Trans	439.0	179	641.8
7/30/2012	Fish	PostFish	79.8	-	-
8/13/2012	Fish	PostFish	5.7	1319	-
8/27/2012	Fish	PostFish	0.8	4208	41.6
9/24/2012	Fish	Mixed	0.0	2868	300.2
6/5/2013	Fish	PreFish	21.5	4247	12.5

6/17/2013	Fish	PreFish	264.8	3552	27.9
7/2/2013	Fish	PreFish	61.3	1629	46.5
7/29/2013	Fish	PostFish	284.3	3353	43.4
8/20/2013	Fish	PostFish	9.1	-	182.4
9/16/2013	Fish	Mixed	0.4	2418	108.9
5/16/2011	No Fish	Mixed	332.0	426	16.9
6/29/2011	No Fish	PreFish	701.3	3695	119.8
7/12/2011	No Fish	Trans	423.0	1392	505.5
8/2/2011	No Fish	PostFish	114.9	4501	700.5
8/30/2011	No Fish	PostFish	516.6	635	335.1
9/27/2011	No Fish	Mixed	403.6	23933	81.5
10/18/2011	No Fish	Mixed	121.0	33157	1893.4
5/16/2012	No Fish	Mixed	17.3	2051	113.7
5/31/2012	No Fish	PreFish	313.6	2127	50.8
6/20/2012	No Fish	PreFish	164.1	3910	73.2
7/2/2012	No Fish	PreFish	384.3	3232	120.9
7/16/2012	No Fish	Trans	326.3	2374	99.4
7/30/2012	No Fish	PostFish	501.4	-	-
8/13/2012	No Fish	PostFish	827.4	333	-
8/27/2012	No Fish	PostFish	513.5	912	2251.9
9/24/2012	No Fish	Mixed	47.9	3221	597.5
6/5/2013	No Fish	PreFish	86.3	4717	14.5
6/17/2013	No Fish	PreFish	261.2	1654	229.0
7/2/2013	No Fish	PreFish	87.4	4972	63.6
7/29/2013	No Fish	PostFish	552.0	1842	1170.0
8/20/2013	No Fish	PostFish	252.2	-	447.3
9/16/2013	No Fish	Mixed	466.7	5503	811.5

Supplementary References

1. Taipale, S., Kankaala, P., Hämäläinen, H., Jones, R.I. Seasonal shifts in the diet of lake zooplankton revealed by phospholipid fatty acid analysis. *Freshw. Biol.* **54,** 90–104 (2009).
2. Taipale, S., Kankaala, P., Tiirola, M. & Jones, R. I. Whole-lake dissolved inorganic ^{13}C additions reveal seasonal shifts in zooplankton diet. *Ecology* **89,** 463–474 (2008).
3. Lane, D. J. in *Nucleic acid Tech. Bact. Syst.* (Stackebrandt, E. & Goodfellow, M.) 115–175 (John Wiley & Sons, 1991).
4. Kolb, S. & Knief, C. Quantitative detection of methanotrophs in soil by novel pmoA-targeted real-time PCR assays. *Appl. Environ. Microbiol.* **69,** 2423–2429 (2003).
5. Rahman, M., Crombie, A. & Chen, Y. Environmental distribution and abundance of the facultative methanotroph Methylocella. *Multidiscip. J. Microb. Ecol.* **5,** 1061–6 (2010).
6. Sharp, C., Stott, M. & Dunfield, P. Detection of autotrophic verrucomicrobial methanotrophs in a geothermal environment using stable isotope probing. *Front. Microbiol.* **3,** 303 (2012).
